# Effect of High Ratio of n-6/n-3 PUFAs on Depression: A Meta-Analysis of Prospective Studies

**DOI:** 10.3389/fnut.2022.889576

**Published:** 2022-05-19

**Authors:** Yuanyuan Wang, Lirong Dong, Da Pan, Dengfeng Xu, Yifei Lu, Shiyu Yin, Shaokang Wang, Hui Xia, Wang Liao, Guiju Sun

**Affiliations:** ^1^Key Laboratory of Environmental Medicine and Engineering of Ministry of Education, Department of Nutrition and Food Hygiene, School of Public Health, Southeast University, Nanjing, China; ^2^Department of Integrated Service and Management, Jiangsu Provincial Center for Disease Control and Prevention, Nanjing, China; ^3^China-DRIs Expert Committee on Macronutrients, Beijing, China

**Keywords:** depression, n-3, n-6, polyunsaturated fatty acids, prospective study

## Abstract

**Objective:**

The aim of this systematic review and meta-analysis was to examine the association between high ratio of n-6/n-3 polyunsaturated fatty acids (PUFAs) and depression.

**Methods:**

The authors conducted a meta-analysis of research articles on the association of high ratio of n-6/n-3 PUFAs with the risk of depression published in the online article database on PubMed, Embase, Cochrane library as of December 2021. Pooled odds ratios (OR) were calculated using random effects models. Publication bias was assessed visually by funnel plots and statistically by the Egger’s and Begg’s tests.

**Results:**

Finally, 12 studies included in this systematic review and meta-analysis with a total of 66,317 participants (including 4,173 individuals with depression condition). The pooled results showed that high ratio of n-6/n-3 PUFAs might be positively associated with depression [OR = 1.21, 95% confidence intervals (CIs): 1.04∼1.41]. The I^2^ test indicated that there was a substantial statistical heterogeneity across the included studies (*I*^2^ = 54.38%, *P* = 0.01). Subgroup analysis showed that high ratio of n-6/n-3 PUFAs in blood had no significant association with depression (OR = 1.15, 95%CI: 0.88∼1.50), while high ratio of n-6/n-3 PUFAs in dietary supplements was positively associated with depression (OR = 1.32, 95%CI: 1.16∼1.51).

**Conclusion:**

This meta-analysis confirmed the association between high ratio of n-6/n-3 PUFAs and the risk of depression. High ratio of n-6/n-3 PUFAs in dietary supplementation was positively associated with depression, but had no significant association in the blood. This study suggested that lowering the dietary intake of the ratio of n-6/n-3 PUFAs would be beneficial in the prevention of depression.

## Introduction

Depression is a common mental illness that involves a complex interplay of social, psychological and biological factors. A continuous and prolonged depressed mood is the main clinical feature (1). Depression can have a profound impact on all aspects of life with researches showing a strong link between depression and health, including tuberculosis and cardiovascular disease (2, 3). In addition, it can disrupt sleep and appetite (4). Depression is reported to be the leading cause of disability worldwide and contributes significantly to the global burden of disease (5–7). According to the World Health Organization (WHO), an estimated 5% of adults worldwide suffer from depression (1). The prevalence of depression in the United States was between 5% and 10% and may be higher in some specific settings (8). Meanwhile, the latest results from the China Mental Health Survey indicated that the prevalence of depression in China was 3.6%, with women being more likely than men at all stages (9). Unfortunately, mental illnesses like depression are getting worse. One of the main reasons is the impact of the COVID-19 pandemic on the mental health of the global population (10, 11). How to prevent and treat depression has become a major area of research for researchers worldwide. Positive effects on depressive symptoms or depression through dietary changes have been demonstrated in many observational and clinical studies (12–15).

The n-3 polyunsaturated fatty acids (PUFAs) and n-6 PUFAs are important fatty acids required by the human body and mainly provided by dietary intake. They are named due to the presence of the first unsaturated bond in the third and sixth positions of the methyl end of the carbon chain, respectively. N-3 PUFAs mainly include alpha-linolenic acid (ALA), eicosapentaenoic acid (EPA) and docosahexaenoic acid (DHA), while n-6 PUFAs mainly include linoleic acid (LA) and arachidonic acid (AA). Recent articles have shown that n-3 PUFAs have myriad health benefits on cardiovascular disease, diabetes, cancer, depression and various mental disorders, age-related cognitive decline, periodontal disease and rheumatoid arthritis (16). Since n-3 and n-6 fatty acids can be converted to share the same family of enzymes, there is competitive metabolism between the n-3 and n-6 fatty acid families (17). Therefore, seeking the optimal balance of the ratio of n-6 to n-3 PUFAs seems to have more health benefits. However, the results of the effects of different ratios of n-6/n-3 PUFAs on depression are inconsistent. Some studies showed that high ratio of n-6/n-3 PUFAs was not associated with depression. A French study on the association between n-3 PUFAs and depression found that n-6/n-3 ratio was not associated with depression, either from cross-sectional data or from cohort data (18). However, there are studies that hold the opposite opinion. An article prospectively examining depression in 54,632 US women from the Nurses’ Health Study found that the risk of depression decreased as the n-3/n-6 ratio increased, while intake of long-chain n-3 fatty acids from fish was not associated with risk of depression (19). They suggested that it was the n-6/n-3 ratio rather than n-3 fatty acids alone that played a role in depression (19, 20). Besides, in a meta-analysis, a higher ratio of n-6/n-3 PUFAs was positively associated with depression (21). However, this meta-analysis only focused on the gestational population and the results could not be extrapolated to the whole population, which would affect the overall prevention and treatment policy of depression. Therefore, examining the relationship between the ratio of n-6/n-3 PUFAs and depression in the whole population has become the focus of this study, which can provide a scientific basis for the primary and secondary prevention of depression.

## Materials and Methods

### Search Strategy

We conducted a systematic search on the databases such as PubMed, Embase, and Cochrane library up to December 2021. We used the following key words for the literature search: (“depression” or “depressive symptoms” or “depressive symptom” or “symptom, depressive” or “symptoms, depressive” or “emotional depression” or “depression, emotional”) AND (“n-6: n-3 fatty acid ratio” or “n-6/n-3 PUFAs” or “n-3 PUFAs” or “omega-3 fatty acid” or “n-6 PUFAs” or “omega-6 fatty acid” or “α-linolenic acid” or “DHA” or “EPA” or “arachidonic acid” or “linoleic acid” or “fish oil” or “fish”). All indexed studies were retrieved and the reference list of identified publications was reviewed for other relevant studies.

### Eligibility Criteria

The criteria of the inclusion in this study were as follows: (1) this study was limited to English-language publications; (2) studies included only prospective cohort studies; (3) human studies; (4) participants had a clear ratio of n-6/n-3 PUFAs by dietary supplementation or biochemical testing for fatty acids; (5) only original studies were included in this study while those studies that were non-original studies (reviews, editorials or commentaries), abstracts, unpublished studies and duplicate studies were excluded.

### Data Extraction

In this study, dietary intake and blood levels of n-3 and n-6 PUFAs were considered the primary exposures, and risk of depression was considered the primary outcome. Adjusted effect sizes were extracted where available. Only total n-3 and n-6 PUFAs content was chosen to be calculated in this study. Risk estimates (ORs or HRs or RRs) were pooled prior to data analysis.

In addition, the following characteristics of eligible articles were extracted: name of first author, date of publication, source of study, numbers of participants completing the study, length of cohort study, type of exposure (dietary intake, blood fatty acids), outcome of interest (depressive status), reported risk assessment related to depression [including ORs, RRs, HRs and their 95% confidence intervals (95%CIs)]. Each step was assessed by two independent investigators. In the event of inconsistent results, the final decision was made primarily by the investigators.

### Quality Assessment of Studies

The Newcastle-Ottawa Scale (NOS) was used to determine the quality of included articles. According to the STAR scoring system, each prospective study is awarded a maximum of nine points based on criteria in three domains: selection (maximum 4 points), comparability (maximum 2 points) and assessment of results (maximum 3 points). According to the NOS, one to three stars indicate low quality, four to six stars indicate moderate quality and seven to nine stars indicate high quality. Quality was assessed independently by the two authors and any disagreements were resolved through discussion.

### Statistical Analysis

In this meta-analysis of prospective cohort studies, log ORs and standard errors (SEs) were calculated using ORs, RRs and HRs and their 95% CIs. At first, a fixed-effects model was used to drive the overall effect sizes. If there was significant between-studies heterogeneity, the random-effects model (DerSimonian–Laird) was applied as an alternative. Cochrane Q test and I^2^ were used to measure potential sources of heterogeneity across studies. In this study, I^2^ > 50 was used as an indicator of heterogeneity among studies. Subgroup analyses were performed using random effects models for the following criteria: source of n-3 and n-6 PUFAs (food intake or blood), quality assessment score (> 6/ ≤ 6) and covariates such as gender, BMI, energy, smoking, alcohol consumption.

Sensitivity analysis was performed to elucidate the stability of findings and to ascertain whether the final pooled effect sizes were affected by a single or several publications. In addition, plausible publication bias was specified visually by funnel plot and confirmed by the statistical evidence of Egger’s test. Data analyses were performed on Stata version 16.0 (Stata Corp., College Station, TX). P values of two sides were considered significant at the level of <0.05.

## Results

### Literature Search

**FIGURE 1:**
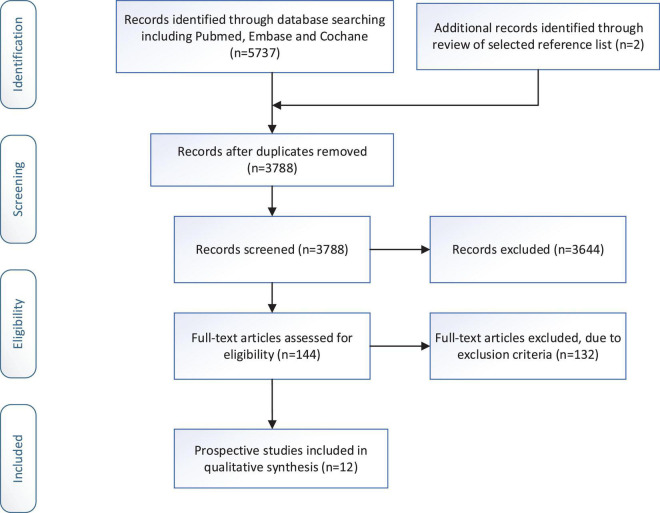
Literature search flow chart.

We identified 5,739 articles from the original search by keywords. Of these, 1,951 articles were excluded because they were duplicates. Then, 3,644 articles were excluded because there was no relevant study design (non-prospective cohort study) or non-human studies. 144 articles were left for full-text examination. Through checking the full-text, twelve eligible papers with 66,317 participants (including 4,173 individuals with depression condition) were included in the current meta-analysis. The process of the literature search is presented in Figure 1.

### Study Characteristics

Twelve prospective studies were finally selected for inclusion in the current systematic review. Characteristics of each study are provided in Table 1. Publication date varied between 2006 and 2021. Four of the included studies were conducted in Europe (18, 22–24), four in Asia (25–28), and four in America (19, 20, 29, 30). Six studies were conducted on women, one study was conducted on men, while five studies included both genders. None of the studies considered the gender-specific association between n-6/n-3 and the risk of depression. Six of the included studies were conducted on dietary intake, while six of the included studies were conducted on blood biochemistry tests. The studies’ sample size ranged from 71 to 54,632. In total, 66,317 individuals, with depression (*n* = 4173) were entered in the current systematic review.

**TABLE 1:** Characteristics of the included studies.

Study source	Population	Follow-up duration (years)	Participants/case	Exposure measurement	Odds ratio (95%CI)
Miyake, et al. (25)	The Osaka Maternal and Child Health Study in Japan	2–9 months postpartum	865/121	Food intake n-6: LA, AA n-3: ALA, EPA, DHA	1.03 (0.6∼1.82)
Lucas, et al. (19)	Women from the Nurses’ Health Study in United States	10	54632/2823	Food intake n-6: LA, AA n-3: ALA, EPA, DHA	1.35 (1.11∼1.64)
Ruusunen, et al. (22)	Kuopio Ischaemic Heart Disease Risk Factor (KIHD) Study cohort in Finland	18	2077/46	Blood n-6: LA, AA n-3: ALA, EPA, DHA, DPA	0.97 (0.49∼2.00)
Kesse-Guyot, et al. (18)	Participants from the Supplementation with Antioxidant Vitamins and Minerals (SU.VI.MAX) Study in France	10.8	1235/140	Food intake n-6: LA, AA n-3: ALA, EPA, DPA, DHA	0.98 (0.58∼1.65)
da Rocha and Kac (20)	A prospective observational cohort of pregnant women in Brazil	at least 30 days post-partum	106/28	Food intake n-6: total n-6 PUFA n-3: total n-3 PUFA	2.50 (1.21∼5.14)
Chong, et al. (26)	Mothers from the Growing Up in Singapore Toward healthy Outcomes (GUSTO) mother-offspring cohort study in Singapore	3 mothers postgratum	698/72	Blood n-6: AA n-3: DHA, EPA, DPA	0.70 (0.09∼5.14)
Matsuoka, et al. (28)	Participants from Japan Public Health Center-based Prospective Study (JPHC Study)	10	1181/99	Food intake n-6: LA, AA n-3: ALA, EPA, DHA, DPA	1.16 (0.64∼2.08)
Pinto, et al. (23)	Pregnant women from a prospective observational cohort in Brazil	30–36 gestational weeks	138/24	Blood n-6: LA, γ linolenic acid, AA, eicosatrienoic acid, docosatetraenoic acid, doco- sapentaenoic acid n-3: ALA, EPA, DPA, DHA	1.40 (1.09∼1.79)
Horikawa, et al. (27)	Participants from the National Institute for Longevity Sciences-Longitudinal Study of Aging (NILS-LSA) in Japan	8.1	2335/515	Food intake n-6: AA, LA n-3: DHA, EPA, ALA	1.36 (1.10∼1.69)
Hoge, et al. (29)	Pregnant women from a prospective observational cohort in Belgian	one year after delivery	71/17	Blood n-6: total n-6 PUFA n-3: total n-3 PUFA	2.31 (1.20∼4.45)
Thesing et al. (24)	Participants from the Netherlands Study of Depression and Anxiety and the Depression Evaluation Longitudinal Therapy Assessment studies in Netherlands	8	474/165	Blood n-6: LA, γ linolenic acid, Eicosadienoic acid, Homogamma-Linolenic Acid, AA, Docosadienoic acid, Docosatetraenoic acid, Docosapentaenoic acid n-3: ALA, EPA, DPA, DHA	0.90 (0.76∼1.06)
Mongan, et al. (30)	Participants from the Avon Longitudinal Study of Parents and Children (ALSPAC) in United Kingdom	7	2505/157	Blood n-6: total n-6 PUFA n-3: total n-3 PUFA	1.02 (0.79∼1.32)

The risk assessment of bias for each study through NOS was shown in Table 2. In terms of selection of populations, the exposed cohort in most studies were underrepresented and only represented a certain group of people. Whereas the non-exposed population was from the same population as the exposed population. Besides, all studies were highly comparable between groups. As for outcome measures, most of the studies had strict outcome measures.

**TABLE 2:** Quality assessment of the included cohort studies.

Study design	Selection (✰ ✰ ✰ ✰)	Comparability (✰ ✰)	Exposure or Outcome (✰ ✰ ✰)	Stars	Quality scores
Cohort studies	1) Representativeness of the exposed cohort? **✰**	1) Study controls for the most important factor? **✰**	1) How to ascertain outcome? **✰**	**✰ ✰ ✰ ✰ ✰** **✰ ✰ ✰ ✰** (9)	High quality: 8–9 stars, Moderate quality: 6–7 stars.
	2) Selection of the non-exposed cohort? **✰**	2) Study controls for any additional factors? **✰**	a) Independent blindness		
	3) Evaluating exposure? **✰**		b) Record linkage		
	4) Outcomes of interest were not present at study start? **✰**		2) Follow-up till outcomes happened? **✰**		
			3) Adequacy of follow up? **✰**		
Included cohort studies					
Miyake, et al. (25)	1) × : female cohort, 2) **✰**, 3) × , 4) **✰**	1) **✰**, 2) **✰**	1) × , 2) **✰**, 3) **✰**	**✰ ✰ ✰ ✰ ✰ ✰**	Moderate
Lucas, et al. (19)	1) × : female cohort, 2) **✰**, 3) **✰**, 4) **✰**	1) **✰**, 2) **✰**	1) **✰**, 2) **✰**, 3) **✰**	**✰ ✰ ✰ ✰ ✰** **✰ ✰ ✰**	High
Ruusunen, et al. (22)	1) × : male cohort; 2) **✰**, 3) **✰**, 4) **✰**	1) **✰**; 2) **✰**	1) **✰**, 2) **✰**, 3) **✰**	**✰ ✰ ✰ ✰ ✰** **✰ ✰ ✰**	High
Kesse-Guyot, et al. (18)	1) **✰**, 2) **✰**, 3) × , 4) × : no statement	1) **✰**; 2) **✰**	1) × , 2) **✰**, 3) **✰**	**✰ ✰ ✰ ✰ ✰ ✰**	Moderate
da Rocha and Kac (20)	1) × : female cohort, 2)**✰**, 3) **✰**, 4) × : no statement	1) **✰**; 2) **✰**	1) × , 2) **✰**, 3) **✰**	**✰ ✰ ✰ ✰ ✰ ✰**	Moderate
Chong, et al. (26)	1) × : female cohort, 2) **✰**, 3) **✰**, 4) **✰**	1) **✰**, 2) **✰**	1) ×, 2) **✰**, 3) **✰**	**✰ ✰ ✰ ✰ ✰** **✰ ✰**	High
Matsuoka, et al. (28)	1) **✰**, 2) **✰**, 3) **✰**, 4) × : no statement	1) **✰**, 2) **✰**	1) **✰**, 2) **✰**, 3) **✰**	**✰ ✰ ✰ ✰ ✰** **✰ ✰ ✰**	High
Pinto, et al. (23)	1) × : female cohort, 2) **✰**, 3) **✰**, 4) **✰**	1) **✰**, 2) **✰**	1) **✰**, 2) **✰**, 3) **✰**	**✰ ✰ ✰ ✰ ✰** **✰ ✰ ✰**	High
Horikawa et al. (27)	1) **✰**, 2) **✰**, 3) **✰**, 4) **✰**	1) **✰**, 2) **✰**	1) × , 2) **✰**, 3) **✰**	**✰ ✰ ✰ ✰ ✰** **✰ ✰ ✰**	High
Hoge et al. (29)	1) × : female cohort, 2)**✰**, 3) **✰**, 4) × : no statement	1) **✰**, 2) **✰**	1) × , 2) **✰**, 3) **✰**	**✰ ✰ ✰ ✰ ✰ ✰**	Moderate
Thesing et al. (24)	1) **✰**, 2) **✰**, 3) **✰**, 4) **✰**	1) **✰**, 2) **✰**	1) × , 2) **✰**, 3) **✰**	**✰ ✰ ✰ ✰ ✰** **✰ ✰ ✰**	High
Mongan et al. (30)	1) **✰**, 2) **✰**, 3) **✰**, 4) **✰**	1) **✰**, 2) **✰**	1) × , 2) **✰**, 3) **✰**	**✰ ✰ ✰ ✰ ✰** **✰ ✰ ✰**	High

Ultimately, we found that four of the twelve included in this meta-analysis were of moderate quality and eight were of high quality.

### Effect of High Ratio of n-6/n-3 PUFAs on Depression

**FIGURE 2:**
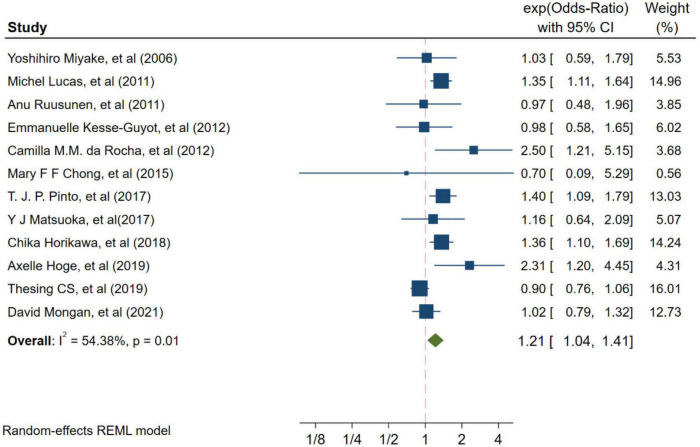
Odds ratios of depression for highest vs. lowest category of ratio of n-6/n-3 PUFAs. Overall odds ratios calculated with random effects model.

**FIGURE 3:**
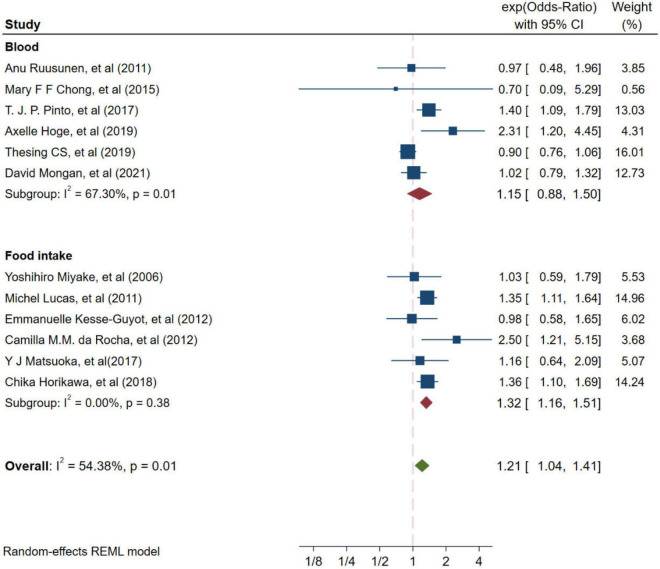
Odds ratios of depression for highest vs. lowest category of ratio of n-6/n-3 PUFAs from different resources. Overall odds ratios calculated with random effects model.

Twelve studies from independent cohorts reported an association between high ratio of n-6/n-3 PUFAs and risk of depression, with 66,317 participants and 4,173 depression events. The forest plot of depression is shown in Figure 2. The pooled results from the forest plot showing in Figure 2, demonstrated that high ratio of n-6/n-3 PUFAs was positively associated with depression (OR = 1.21, 95%CI: 1.04∼1.41). The I^2^ test indicated that there was a substantial statistical heterogeneity across the included trials (*I*^2^ = 54.38%, *P* = 0.01). Moreover, as shown in Figure 3, six studies, which detected n-3 and n-6 PUFAs in blood, indicated that high ratio of n-6/n-3 PUFAs had no significant association with depression (OR = 1.15, 95%CI: 0.88∼1.50), with a substantial statistical heterogeneity across the included trials (*I*^2^ = 67.30%, *P* = 0.01). However, six studies conducting on dietary intake showed that high ratio of n-6/n-3 PUFAs was positively associated with depression (OR = 1.32, 95%CI: 1.16∼1.51), with a non-significant heterogeneity between studies (*I*^2^ = 0.00%, *P* = 0.38).

### Sensitivity Analysis and Subgroup Analysis

Sensitivity analysis showed that the effect of high ratio of n-6/n-3 PUFAs on depression was not changed by removing any one of the studies at a time. When studies with one or more high risks of bias were excluded, the overall effect size was not significantly changed for depression.

To more precisely identify the relationship between high ratio of n-6/n-3 PUFAs and depression, we performed further substantification analysis of the screened studies (shown in Table 3). We found that in America and Asia, the high ratio of n-6/n-3 PUFAs showed a significantly increasing effect on depression (OR = 1.44, 95CI%: 1.24∼1.67; OR = 1.29, 95CI%: 1.07∼1.56, respectively), with a substantial statistical heterogeneity across the included trials (I^2^ = 0.00%, P = 0.19; I^2^ = 0.00%, P = 0.72, respectively). However, in European countries, the high ratio of n-6/n-3 PUFAs had no significant effect on depression (OR = 0.94, 95CI%: 0.82∼1.07). The significant positive association with high ratio of n-6/n-3 PUFAs and the risk of depression was observed in pregnant women (OR = 1.53, 95CI%: 1.10∼2.13, I^2^ = 36.46%, P = 0.19), while it was not significant for high ratio of n-6/n-3 PUFAs among studies in healthy people (OR = 1.12, 95CI%: 0.94∼1.32). Additionally, high ratio of n-6/n-3 PUFAs was associated with depression in studies that adjusted for BMI. Besides, the significant positive association was observed in unadjusted model with education, smoke and drink compared with those studies with such adjustment, while there was no significant relationship between high ratio of n-6/n-3 PUFAs and depression in both unadjusted and adjusted model with energy and education.

**TABLE 3:** Subgroup analyses of high ratios of n-6/n-3 PUFAs and risk of depression (highest vs. lowest category).

Subgroup	No. of studies	Odds risk (95% CI)	I^2^%	*P* value
**Regions:**				
America	4	1.44 (1.24∼1.67)	0.00	0.19
Asia	3	1.29 (1.07∼1.56)	0.00	0.72
Europe	4	0.94 (0.82∼1.07)	0.00	0.88
**Population:**				
Pregnant women	5	1.53 (1.10∼2.13)	36.46	0.19
Healthy people	6	1.12 (0.94∼1.32)	56.53	0.03
**Covariate method:**				
Adjustment for age	8	1.15 (0.99∼1.33)	53.18	0.03
No adjustment for age	3	2.24 (1.40∼3.59)	0.00	0.50
Adjustment for BMI	7	1.20 (1.02∼1.40)	22.39	0.45
No adjustment for BMI	4	1.39 (0.96∼2.01)	82.56	0.00
Adjustment for energy	2	1.26 (0.97∼1.65)	22.58	0.26
No adjustment for energy	9	1.22 (1.01∼1.47)	59.93	0.01
Adjustment for education	4	1.07 (0.84∼1.36)	61.91	0.03
No adjustment for education	7	1.32 (1.11∼1.57)	32.47	0.13
Adjustment for smoking	7	1.11 (0.95∼1.30)	51.46	0.05
No adjustment for smoking	4	1.72 (1.18∼2.52)	34.37	0.23
Adjustment for drinking	4	1.07 (0.86∼1.35)	53.31	0.06
No adjustment for drinking	7	1.33 (1.10∼1.63)	43.59	0.09
**Risk expression**				
Hazard/rate ratio	2	1.10 (0.73∼1.65)	88.73	0.00
Odds ratio	7	1.27 (1.02∼1.58)	38.82	0.11
Relative risk	2	1.32 (1.09∼1.59)	0.00	0.37
**Quality assessment**				
High	6	1.17 (0.99∼1.37)	58.12	0.02
Moderate	5	1.49 (0.91∼2.43)	61.58	0.05

### Publication Bias

There was no significant evidence of publication bias as indicated by the results from Begg’s test and Egger’s test for the relationship between high ratio of n-6/n-3 PUFAs and depression (P_Begg_ = 0.451, P_Egger_ = 0.581).

## Discussion

A growing number of researchers recommend that the intake of n-6 PUFAs should be considered alongside n-3 PUFAs (31–33). The current conflicting findings on the relationship between high ratio of n-6/n-3 PUFAs and depression is not conducive to the development of strategies related to the treatment of depression. Therefore, the aim of this meta-analyses was to examine the relationship between high ratio of n-6/n-3 PUFAs and depression in the whole population.

In the final twelve cohort studies included, we concluded that a high ratio of n-6/n-3 PUFAs was indeed positively associated with depression. A study in Japan examining the relationship between n-3 unsaturated fatty acids and the tendency to depression in healthy people showed that 22.1% of people suffered from depressive conditions over an average follow-up of 8.1 years, and that high proportions of n-6/n-3 increased the risk of developing depressive symptoms (27). The results of the maternal population study also showed that a higher ratio of n-6/n-3 PUFAs was associated with a higher risk of depressive symptoms in the first year after delivery (29). Several potential mechanisms have been proposed regarding the association between high ratio of n-6/n-3 PUFAs, one of which is the inflammatory response (34). Depression has been associated with activation of the inflammatory response with this association being bidirectional (35, 36). For some depressed patients, inflammation promotes the onset of depression; meanwhile depression stimulates a greater cytokine response to stress (37). Therefore, depression can be alleviated by decreasing the inflammatory response. EPA and DHA are often considered to have anti-inflammatory effects and may promote the reduction of inflammation. Studies have shown that the anti-inflammatory effects of EPA and DHA were enhanced when the intake of AA was reduced. It indicates that the ratio of n-6/n-3 PUFAs seems to be more sensitive to the inflammatory response and thus to depression (38). A randomized controlled trial revealed that an increased ratio of n-6/n-3 PUFAs was associated with major depression and increased production of pro-inflammatory cytokines in students. This study found that lowering the n-6/n-3 PUFAs ratio resulted in lower anxiety and stimulated reductions in IL-6 and tumor necrosis factor alpha (TNF-α) production, as well as small differences in serum TNF-α (39). However, the interactions regarding n-3 and n-6 fatty acids in the context of inflammation are complex and still need to be justified by a large number of studies. From the public health point of view, lowering the n-6/n-3 PUFAs ratio in the diet and maintaining the dynamic interactions between n-3 and n-6 PUFAs (PUFAs balance), which according to some studies are certainly better indicators of health effects than individual PUFA concentrations, are both relevant for depression prevention (40).

In subgroup analyses, we found that low ratio of dietary-derived n-6/n-3 PUFAs supplementation significantly reduced depression. In a cross-sectional study from Japan, a significant negative association was found between the low ratio of n-3/n-6 PUFAs in the dietary intake of overweight and obese women and depressive symptoms (41). Similar results were found in depression during pregnancy. A study showed that pregnant women with higher than recommended dietary intakes of total fatty acids and high ratio of n-6/n-3 were at higher risk of developing depressive symptoms (42). However, low ratio of blood-derived n-6/n-3 PUFAs in our study did not significantly reduce depression. Some studies have shown that a higher n-6/n-3 PUFAs ratio in the blood was positively associated with depression (21, 43–45). Depression and the n-6/n-3 PUFAs ratio acted together to enhance pro-inflammatory cytokines beyond the contribution provided by either variable alone, and as depressive symptoms increased, higher ratio of n-6/n-3 PUFAs was associated with progressively higher levels of TNF-α and IL-6 (44). The potential reason for this was that the small sample size of ratio of n-6/n-3 PUFAs derived from blood produced higher heterogeneity. Besides, we should consider that dietary intake of PUFAs may not fully reflect the amount of fatty acids in the blood. A prospective study showed that PUFAs determined at baseline in red blood cells and in the diet were differentially associated with cognitive function and cognitive impairment (46). A study from the United States also noted that both plasma EPA and DHA concentrations were significantly predicted by dietary intake of these fatty acids. However, plasma docosapentaenoic acid (DPA) levels were not related to dietary intake of DPA (47). Therefore, our next study sought to explore the relationship between the ratio of n-6/n-3 PUFAs in the diet and blood, clarify the underlying mechanisms of this relationship, and figure out the relationship between the ratio of n-6/n-3 PUFAs in the diet and blood and depression.

## Strengths and Limitations

Current research findings on the relationship between high ratio of n-6/n-3 PUFAs and depression is conflicting, which will influence the formulation of policies related to the prevention and treatment of depression. To our knowledge, this is the first study that focuses on the relationship between high ratio of n-6/n-3 PUFAs and depression. This study included a high-quality grade of cohort studies from a variety of countries, so there is a high degree of confidence in the pooled results. In addition, we conducted a series of subgroup and meta-regression analyses to explore sources of heterogeneity and thus improve the accuracy of the results for the studies of interest. The robustness of our results was supported by sensitivity analyses. There was no significant publication bias in our study through the use of the Begg’s test and the Egger’s test.

However, there are some limitations in the meta-analysis. Firstly, different assessment methods were used in the included studies, mainly derived from fatty acids in dietary intake and blood, but this was resolved by subgroup analysis. Secondly, language bias may have arisen as we excluded articles that were not in English. However, we selected articles covering most of non-English speaking Europe and Asia, with a limited number of cohort studies from other countries. Finally, n-3 and n-6 unsaturated fatty acids were also calculated differently in different studies; for example, some studies included EPA and DHA content as total n-3 unsaturated fatty acid content, which undoubtedly biased the results. Therefore, future studies should be more standardized in their calculation of fatty acids, and it is hoped that large, high-quality, long-term randomized controlled trials will also be conducted to provide more reliable clinical evidence.

## Conclusion

This study had significant public health implications. Our meta-analysis found a positive association between high ratio of n-6/n-3 PUFAs and depression and this positive association was only present in high ratio of n-6/n-3 PUFAs in dietary supplementation but not in blood. This study suggests that lowering the dietary intake of the ratio of n-6/n-3 PUFAs would be beneficial in the prevention of depression.

## Data Availability Statement

The original contributions presented in the study are included in the article/Supplementary Material, further inquiries can be directed to the corresponding author/s.

## Author Contributions

YW designed the study and wrote the manuscript. YW and LD searched and reviewed the relevant trials and collected the data. DP and DX played a role as a consultant. YL and SY helped employ search strategies. HX and WL performed statistical analysis. SW was responsible for the quality assessments for the studies. GS was the corresponding author. All authors contributed to the article and approved the submitted version.

## Conflict of Interest

The authors declare that the research was conducted in the absence of any commercial or financial relationships that could be construed as a potential conflict of interest.

## Publisher’s Note

All claims expressed in this article are solely those of the authors and do not necessarily represent those of their affiliated organizations, or those of the publisher, the editors and the reviewers. Any product that may be evaluated in this article, or claim that may be made by its manufacturer, is not guaranteed or endorsed by the publisher.
